# *Bacteroides fragilis* strain ZY-312 promotes intestinal barrier integrity via upregulating the STAT3 pathway in a radiation-induced intestinal injury mouse model

**DOI:** 10.3389/fnut.2022.1063699

**Published:** 2022-12-15

**Authors:** Qian Zhou, Binhai Shen, Ruo Huang, Hongbin Liu, Wendi Zhang, Mengyao Song, Ke Liu, Xinlong Lin, Shuze Chen, Yangyang Liu, Ye Wang, Fachao Zhi

**Affiliations:** ^1^Department of Gastroenterology, Institute of Gastroenterology of Guangdong Province, Guangdong Provincial Key Laboratory of Gastroenterology, Nanfang Hospital, Southern Medical University, Guangzhou, China; ^2^Guangzhou Zhiyi Biotechnology Co., Ltd., Guangzhou, China

**Keywords:** *Bacteroides fragilis* strain ZY-312, probiotics, radiation-induced intestinal injury, stem cells, goblet cells, tight junction, STAT3 signaling pathway

## Abstract

Radiation-induced intestinal injury is characterized by intestinal barrier impairment. However, the therapeutic effects of probiotics for intestinal epithelial barrier repair in a mouse model of radiation-induced intestinal injury remain unclear. Previously, we isolated a strain of *Bacteroides fragilis* from the feces of a healthy infant and named it as *B. fragilis* strain ZY-312 (*B. fragilis*). In this study, we showed that *B. fragilis* can ameliorate radiation-induced intestinal injury in mice, manifested by decreased weight loss, intestinal length shortening, and intestinal epithelial cell (IEC) shedding. Moreover, we found that *B. fragilis* promoted IEC proliferation, stem cell regeneration, mucus secretion, and tight junction integrity by upregulating the STAT3 signaling pathway, through an experimental verification in *Stat3*^△IEC^ mice (STAT3 defects in intestinal epithelial cells). Thus, the underlying protective mechanism of *B. fragilis* in radiation-induced intestinal injury is related to IEC proliferation, stem cell regeneration, goblet cell secretion, and tight junction repair *via* activation of the STAT3 signaling pathway. In addition, the therapeutic effects of *B. fragilis* were studied to provide new insights into its application as a functional and clinical drug for radiation-induced intestinal injury after radiotherapy.

## Introduction

With the application of radiation delivery techniques in oncotherapy, 70% of cancer patients have benefited from radiotherapy, and 25% of them have become survivors (1, 2). However, gastrointestinal syndrome is a potentially serious complication of radiation therapy. Gastrointestinal syndrome is complex, characterized by gastrointestinal toxicity and radiation-induced intestinal injury. The major symptoms of radiation-induced intestinal injury in humans include diarrhea, abdominal pain, constipation, hematochezia, and weight loss (3). The acute radiation-induced intestinal injury occurs within 3 months, while chronic radiation-induced intestinal injury appears >3 months after radiotherapy (4, 5). It has been reported that 90% of cancer patients receiving abdominal radiotherapy develop radiation-induced intestinal injury within a few weeks (6) and life-threatening systemic infections after 3 months (7), thus compromising the patients’ quality of life. Therefore, it has become an urgent priority to seek effective therapies for side effects caused by radiotherapy.

Due to the fact that small intestinal epithelial cells (SI IECs) are major sensitive sites of abdominal radiotherapy (8), the healthy intestine is inevitably exposed to radiation during radiotherapy for abdominal and pelvic malignant tumors (9). Although the process of intestinal regeneration is initiated after radiation injury, there is also a rapid loss of LGR5 + intestinal stem cells (ISCs) and proliferating progenitor cells (10). The ionizing radiation will cause injury to basal epithelial cells and impede their renewal, leading to histologically detectable alterations of IECs such as decreased intestinal villous height and number, inflammation, bowel wall edema, and fibrin precipitation (11). In addition, manifestations such as diarrhea occur in humans and mice a few weeks after radiation injury due to the atrophy, ulceration, and high permeability of IECs (12). There is currently no effective therapeutic radioprotectant for general clinical application. Some clinical trials have shown that probiotics have the potential therapeutic effects to mitigate intestinal radiation injuries (13). A double-blind, placebo-controlled clinical trial including approximately 500 patients assigned to either a probiotic preparation (VSL#3) or placebo treatment after receiving adjuvant postoperative radiation therapy reported decreased incidence and severity of radiation-induced intestinal injury in the VSL#3 treatment group (14). Moreover, the application of *Lactobacillus rhamnosus* in radiation therapy improved stool consistency and reduced bowel movements and abdominal discomfort (15), suggesting that probiotics have the potential to become radioprotectants for clinical application.

*Bacteroides fragilis* strain ZY-312 (*B. fragilis*), belonging to the genus *Bacteroides*, was isolated from the feces of a healthy infant (16). O’Toole et al. (17) reported that *B. fragilis* has the potential to become a second-generation probiotic with biological applications. Using a metabolic engineering approach, our team previously found that *B. fragilis* mainly existed in the small intestine and colon (18), and promoted intestinal tissue proliferation, thus relieving antibiotic-related diarrhea in a rat model (19). In addition, we reported that *B. fragilis* inhibited intestinal epithelial cell apoptosis in a *Cronobacter sakazakii*-induced neonatal necrotizing enterocolitis model (20), and restored intestinal barrier to prevent diarrhea in a mouse model of *Clostridium difficile* infection (21). This suggests that *B. fragilis* has the potential to promote intestinal epithelium regeneration, thereby relieving diarrhea caused by abdominal radiotherapy. *B. fragilis* performs the probiotic therapeutic roles in intestinal disease. However, the effects and mechanism of *B. fragilis* in radiation-induced intestinal injury remain unclear. In this study, we aimed to evaluate the effects and underlying mechanism of *B. fragilis* in radiation-induced intestinal injury in mice. We hope that our findings will provide new insight into the treatment of the radiation-induced intestinal injury.

## Materials and methods

### Establishment of radiation-induced intestinal injury model

The mouse model of radiation-induced intestinal injury was constructed by previously described methods (22). The C57BL/6 mice and *Stat3*^△IEC^ mice were gavaged with *B. fragilis* (1 × 10^9^ CFU per mouse) or *B. fragilis*-derived capsular polysaccharide (PSA) (50 μg/mouse) for 14 days during the pretreatment stage, then administered to mice with radiation. Radiation was given using the irradiator (MultiRad, Faxitron, USA) at 26 Gy/min with 225 kVp X-rays using a 0.3 mm copper filter. (1) The control group is the following: Mice were treated with phosphate buffer (PBS) orally. (2) TAI group: Mice were treated with PBS, and exposed to 10 Gy total of abdominal irradiation. (3) TBI group: Mice were treated with PBS, and exposed to 4.5 Gy total body irradiation. (4) TAI/TBI + ZY-312 group: Mice were treated with *B. fragilis* (1 × 10^9^ CFU per mouse) through an oral route, dissolved in sterile water in 0.2 ml volume per mice for 15 consecutive days after 4.5 Gy TBI or 10 Gy TAI. All experiments were performed using 6- to 8-week-old sex-matched mice. C57BL/6 mice were purchased from SPF (Beijing) Biotechnology Co., Ltd. (Beijing, China), and STAT3 conditional gene knockout mice (*Stat3*^△IEC^ mice, the primers for gene identification were showed in Supplementary Table 2) were purchased from GemPharmatech Co., Ltd. (Jiangsu, China).

### Culture of *Bacteroides* fragilis strain ZY-312

*Bacteroides fragilis* strain ZY-312 was cultured in 5% fetal bovine serum, 9.5 ml of tryptic soy broth, and 100 μl of passaging solution at 37°C for 24 h under anaerobic conditions.

### Histological stain

The small intestinal tissue of mice was fixed in 4% paraformaldehyde for 24 h, embedded in paraffin, and cut into sections at a thickness of 5 μm (The histopathology associated index of intestine was evaluated though the criterion in Supplementary Table 1). For immunohistochemical staining, the intestinal sections were subjected to deparaffinization, hydration, antigen retrieval, quenching of endogenous peroxidase, and blocking procedures. All slices were then incubated with the primary antibodies against pSTAT3 (CST #9145) and Ki-67 (Abcam, England, Ab16667) at 4°C overnight followed by incubation with biotinylated secondary antibodies for 30 min and visualization using a 3,3′-Diaminobenzidine Kit (ZSGB-BIO, Beijing, China). For immunofluorescence staining for the detection of MUC2 (Abcam, England, Ab272692), Lgr5 (NBP1-28904SS), ZO-1 (Abcam, England, Ab221547), and Claudin-1 (Abcam, England, Ab242370), the colonic sections were processed by the methods described above. Periodic Acid-Schiff stain (PAS) staining (Biossci, China) was performed following the manufacturer’s protocols.

### Small intestinal epithelial cells isolation

The small intestinal was isolated from mice. Then SI was washed with PBS and cut into 5 mm pieces, followed by digestion with buffer (5 mM EDTA and 2 mM dithiothreitol in Hanks balanced salt solution; Sigma, St. Louis, MO, USA) at 37°C for 30 min on a rotating platform. Through the digestion stopping stage with Hanks balanced salt solution, the solution was filtered through a 70 μm cell strainer. Lastly, the filtered solution was centrifuged for 10 min at 400 *g* (4°C) to obtain SI epithelial cells (23).

### Western blot

Western blotting was performed followed by previous protocols (24). The proteins were separated by SDS–PAGE and analyzed by immunoblotting with rabbit polyclonal antiserum to pSTAT3 (CST #9145, Danvers, MA, USA), STAT3 (CST #4904), PCNA (CST #13110), P38 (CST #9212), pmTOR (CST #4511), mTOR (CST #4511), pJAK2 (CST #4695), pNF-KB (CST#4370), Glyceraldehyde-3-phosphate dehydrogenase (GAPDH) (CST #5174), ZO-1 (Proteintech 21773-1-AP), Claudin-1 (Proteintech 13050-1-AP), MUC2 (Abcam, England, 272692-10), Lgr5 (Abcam, England, 75850) antibodies were used to measure colonic epithelial protein expression.

### 16S rRNA analysis

The fecal microbiota composition of mice was determined by 16S rRNA gene amplification. Briefly, TGuide S96 Magnetic Soil/Stool DNA Kit (Tiangen Biotech Co., Ltd., Beijing, China) was used to extract genomic DNA from feces. DNA concentration and integrity were measured by a microplate reader (GeneCompang Limited, synergy HTX, United States) and agarose gel electrophoresis, respectively. The 16S rRNA gene V3–V4 region was amplified from the genomic DNA in a 25 μl reaction using the universal bacterial primers: (338F, 50–ACTCCTACGGGAGGCAGCA–30, and 806R, 50–GGACTACHVGGGTWTCTAAT–30). The PCR products were quantified by using a NanoDrop 2000 spectrophotometer (Thermo Fisher Scientific, Waltham, MA, United States) and purified with Monarch^®^ DNA gel Extraction Kit (New England Biolabs, USA). Sequencing was performed on an Illumina NovaSeq 6000 with two paired-end read cycles of 250 bases each (Illumina Inc., San Diego, CA, United States; Biomarker Co., Ltd., Beijing, China). The Trimmomatic software v0.33 was used to analyze the raw data. After trimming, amplicon sequence variants (ASVs) were generated by using USEARCH 10.0 with a 97% similarity cutoff. The representative read of each ASV was selected by using the Quantitative Insights into Microbial Ecology (QIIME) package. All representative reads were annotated and blasted against the Silva database (Version 132) using the Ribosomal Database Project (RDP) classifier (confidence threshold was 70%). The microbial richness and diversity in fecal content samples were estimated using the alpha diversity that includes the Shannon index. The UniFrac distance matrix performed by QIIME software was used for the unweighted UniFrac Principal coordinates analysis (PCoA), and phylogenetic tree construction. Linear discriminant analysis effect size (LEfSe) based linear discriminant analysis (LDA) and cladogram were generated to assess differentially abundant microbial taxa. The 16S rRNA gene amplicon sequencing and analysis were conducted by Biomarker Technologies Co., Ltd. (Beijing, China).

### Statistical analysis

Statistical analyses were performed using GraphPad Prism 8.0 (GraphPad Software). All variables are expressed as Means ± SEM, as noted in the figure legends. Two-way ANOVA was used to compare body weights. The one-way ANOVA with Tukey’s tests was used for comparisons among three or more groups. While non-parametric data were analyzed with a Mann–Whitney *U*-test. *P* < 0.05 was considered as significance.

## Results

### *Bacteroides fragilis* alleviated radiation-induced intestinal injury in mice through *Bacteroides fragilis*-derived polysaccharide

**FIGURE 1:**
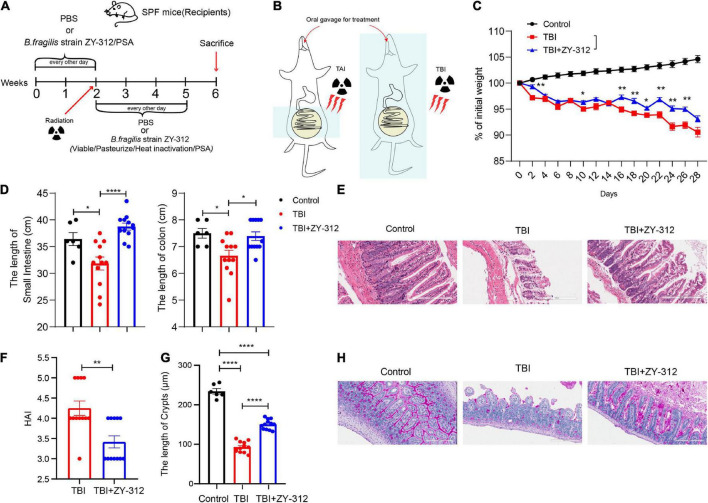
*Bacteroides fragilis* alleviated total body radiation-induced intestinal injury in mice. **(A,B)** The model of radiation-induced intestinal injury in mice. **(C)** The *p*-value of TAI group vs. TAI + ZY-312 group in percent (%) of weight loss is following: percent (%) of weight loss *p*-value (TBI group vs. TBI + ZY-312 group), Day2 0.0025, Day10 0.0483, Day16 0.0064, Day18 0.0017, Day20 0.0236, Day22 0.0017, Day24 0.0024, and Day26 0.0020. **(D)** The *p*-value between groups are following: *p*-value the small intestine length the colon length (Control group vs. TBI group) 0.0261 0.0263 (Control group vs. TBI + ZY-312 group) 0.3468 0.9326 (TBI group vs. TBI + ZY-312 group) <0.0001 and 0.0171. **(E)** The histopathology- associated index (HAI) between groups. **(F)** The *p*-value between groups are following: *p*-value HAI SI crypts (Control group vs. TBI group)/<0.0001 (Control group vs. TBI + ZY-312 group)/<0.0001 (TBI group vs. TBI + ZY-312 group) 0.0017 and <0.0001. **(G)** The length of SI crypts between groups. **(H)** Periodic acid-Schiff stain (PAS) staining of SI. Control group (*N* = 6), TBI group (*N* = 12), TBI + ZY-312 group (*N* = 12). N, the number of mice. The data are presented as mean ± SEM, and *p < 0.05, **p < 0.01, **p < 0.001, **p <0.0001.

**FIGURE 2:**
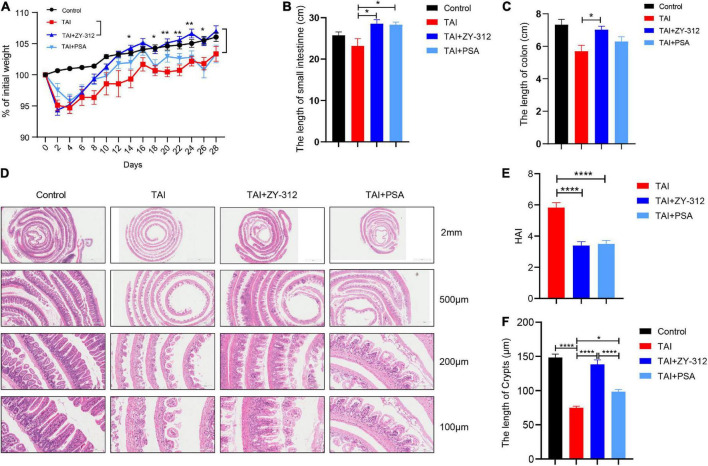
*Bacteroides fragilis* alleviated abdominal radiation-induced intestinal injury in mice through *B. fragilis*-derived polysaccharide (PSA). **(A)** The *p*-value between groups are following: percent (%) of weight loss *p*-value (TAI group vs. TAI + ZY-312 group), Day14 0.0323, Day18 0.0254, Day20 0.0014, Day22 0.0017, Day24 0.0027, and Day26 0.0431. **(B)** The *p*-value between groups are following: *p*-value the small intestine length (TAI group vs. TAI + ZY-312 group) 0.0149 and (TAI group vs. TAI + PSA group) 0.0202. **(C)** The *p*-value between groups are following: *p*-value the colon length (TAI group vs. TAI + ZY-312 group) 0.0319. **(D)** The histopathology of small intestinal at different magnifications. **(E)** The *p*-value between groups are following: *p*-value HAI (TAI group vs. TAI + ZY-312 group) <0.0001 and (TAI group vs. TAI + PSA group) <0.0001. **(F)** The *p*-value between groups are following: *p*-value SI crypts (Control group vs. TAI group) <0.0001, (TAI group vs. TAI + ZY-312 group) <0.0001, (TAI group vs. TAI + PSA group) 0.014, and (TAI + ZY-312 group vs. TAI + PSA group) <0.0001. The data are presented as mean ± SEM, and *p < 0.05, **p < 0.01, **p < 0.001, **p <0.0001.

To evaluate the effect of *B. fragilis* strain ZY-312 on radiation-induced intestinal injury, we constructed the corresponding mouse model for total body radiation (TBI) and administered the mice with *B. fragilis* orally (Figures 1A,B). Compared with the TBI group, mice receiving *B. fragilis* (TBI + ZY-312 group) had significantly lower weight loss (Figure 1C) and slower SI and colon length shortening (Figure 1D). To explore the effect of *B. fragilis* on the IECs, the histopathological results revealed that *B. fragilis* supplementation attenuated SI epithelial cell damage (Figure 1E), reduced overall HAI, and resulted in longer lengths of SI crypts (Figure 1F). Furthermore, we found that *B. fragilis* promoted SI mucus secretion in radiation-induced intestinal injury (Figure 1G). Concurrently, we constructed a radiation-induced intestinal injury mouse model for total abdominal radiation (TAI) and administered the mice with *B. fragilis* and *B. fragilis*-derived capsular polysaccharide (PSA) (Figures 1A,B). Compared with the TAI group, mice receiving *B. fragilis* (TAI + ZY-312 group) had significantly lower weight loss (Figure 2A) and slower intestinal (SI and colon) length shortening (Figures 2B–C). To explore the effect of *B. fragilis* and PSA on the IECs, the histopathological results revealed that *B. fragilis* supplementation and PSA obviously attenuated SI epithelial cell damage (Figure 2D), reduced overall histopathology-associated index (HAI) (Figure 2E), and resulted in longer lengths of SI crypts (Figure 2F). Thus, *B. fragilis* may alleviate abdominal radiation-induced intestinal injury in mice through *B. fragilis*-derived PSA. And it suggested that *B. fragilis* promoted the integrity of SI epithelium to alleviate radiation-induced intestinal injury in mice.

### *Bacteroides fragilis* promoted stem cell and goblet cell regeneration and accelerated tight junction repair

**FIGURE 3:**
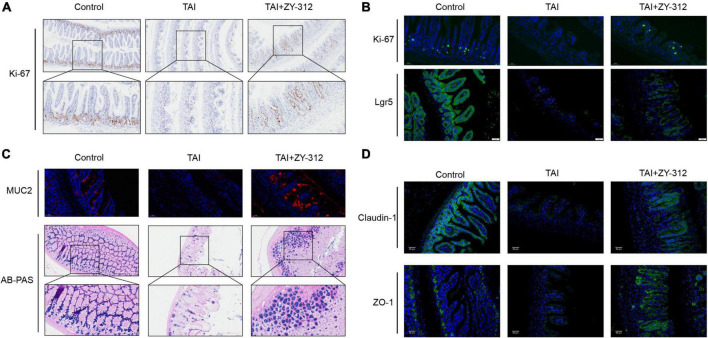
*Bacteroides fragilis* promoted intestinal epithelial cell proliferation, stem cell regeneration, goblet cell secretion, and tight junction repair. **(A)** The immunohistochemical staining of Ki-67 in slower intestinal (SI) between groups. **(B)** The immunofluorescence staining of Ki-67 and Lgr5. **(C)** The immunofluorescence and Alcian blue-periodic acid-Schiff staining (AB-PAS) of SI goblet cells. **(D)** The immunofluorescence staining of Claudin-1 and ZO-1.

Leucine-rich repeat-containing G-protein coupled receptor 5 (LGR5+) ISCs cells are considered active multipotent intestinal stem cells because they have the capacity to divide and differentiate into different types of intestinal epithelial cells, such as goblet cells and enterocytes (11). Thus the degree of stem cell injury is an important indicator for damage repair after radiation. In addition, intestinal epithelial tight junctions, including zonula occludens-1 (ZO-1), occludins, and claudins, are also highly sensitive to ionizing radiation with the disruption of IECs (25). Next, we attempted to determine whether *B. fragilis* has the capacity to alleviate the destruction of SI IECs after radiation. *B. fragilis* was found to promote SI IECs proliferation (Figure 3A). Regarding the types of IECs affected, the small intestinal histological results showed that *B. fragilis* promoted stem cell proliferation (Figure 3B), goblet cell secretion (Figure 3C), and tight junction protein expression (Figure 3D). This indicated that *B. fragilis* promoted stem cell and goblet cell regeneration, and accelerated tight junction repair in radiation-induced intestinal injury in mice.

### *Bacteroides fragilis* did not significantly alter the intestinal microbiota to relieve radiation-induced intestinal injury in mice

**FIGURE 4:**
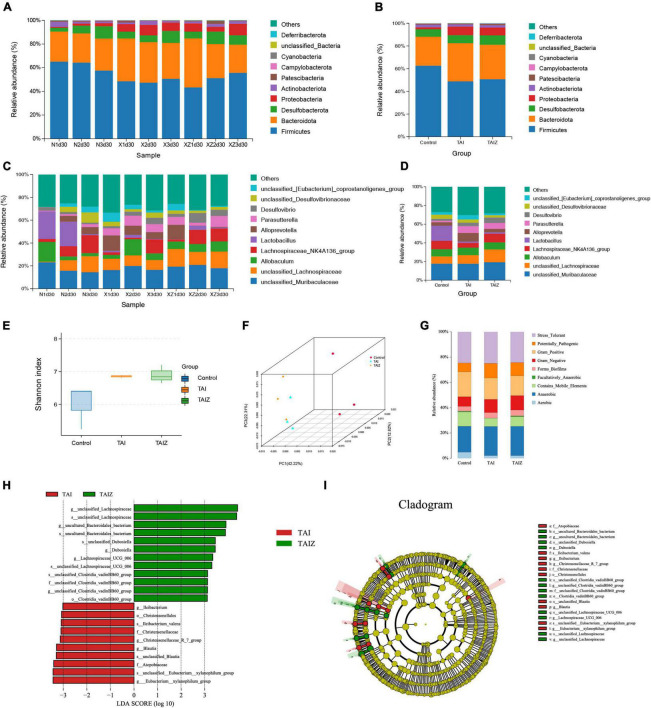
*Bacteroides fragilis* did not significantly alter intestinal microbiota to relieve radiation-induced intestinal injury in mice. **(A,B)** The phylum level of microbiota composition and comparison of microbiota in mice between samples and groups. **(C,D)** The genus level of microbiota composition and comparison of microbiota in mice between samples and groups. **(E)** The abundance and diversity of microbiota (Shannon index) in mice. **(F)** The principal coordinate analysis of microbiota. **(G)** The bug base analysis of microbiota. **(H)** The Linear discriminant analysis effect size (LEfSe) analysis of differential bacteria in slower intestinal (SI) meeting a significant linear discriminant analysis (LDA) threshold value of >3.5 between groups with different levels. **(I)** The cladogram based on LEfSe shows differential bacteria of the gut microbiota between groups with different levels. Nd30, control group; xd30 and TAI, TAI group; xzd30 and TAIZ, TAI + ZY-312 group.

Certain types of intestinal microbiota reside in the mucus layer (26), and mucus has the capacity of resisting pathogenic bacteria in colitis (27). *B. fragilis* has a positive influence on mucus secretion, which suggests that it may alter the intestinal microbiota, thus relieving radiation-induced intestinal injury in mice. Hence we analyzed intestinal microbiota by 16S rRNA gene sequencing. The main intestinal microbiota phyla were *Firmicutes, Bacteroidetes, and Proteobacteria*. And the microbial community bar plot showed the relative abundance of phylum *Firmicutes* increased and that of phylum *Bacteroidetes* decreased gradually in the TAI + ZY-312 group compared to the TAI group, although there was no statistically significant difference (Figures 4A,B). At the genus level, the genera of the *Lachnospiraceae*_NK4A136_group producing butyrate increased in the TAI + ZY-312 group compared to the TAI group, despite it did not show a statistically significant difference (Figures 4C,D). Furthermore, we analyzed the microbiota diversity (Shannon), and conducted principal component analysis (PCA), which reflects the difference in gut microbiota between and within groups. We found that the microbiota diversity between the TAI and TAI + ZY-312 groups did not change significantly (Figures 4E,F). And the BugBase results showed that the abundance of anaerobic microbiota was increased in the TAI and TAI + ZY-312 groups compared to the control group (Figure 4G). LEfSe and phylogenetic tree are analytical methods that could screen the statistical difference biomarker among different groups. The LEfSe results showed that the abundance of the *Clostridiales*_vadinBB60_group was increased in the TAI + ZY-312 group (Figures 4H,I). It indicated that *B. fragilis* participated in modulating intestinal microbiota, although it did not significantly alter the intestinal microbiota in microbiota abundance and diversity.

### *Bacteroides fragilis* upregulated STAT3 signaling pathway in intestinal epithelial cells

**FIGURE 5:**
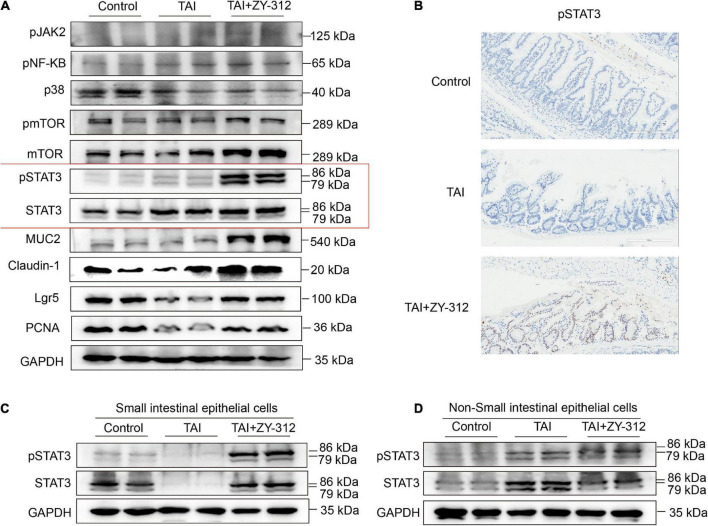
*Bacteroides fragilis* upregulated the signal transducer and activator of transcription 3 (STAT3) signaling pathway in intestinal epithelial cells. **(A)** The western blot analysis of pJAK2, pNF-KB, p38, pmTOR, mTOR, pSTAT3, STAT3, MUC2, Claudin-1, Lgr5, and PCNA in slower intestinal (SI) tissue between groups. GAPDH was regarded as an internal reference. **(B)** The immunohistochemical staining of pSTAT3 in SI between groups. **(C)** The western blot analysis of pSTAT3, STAT3 in SI intestinal epithelial cells (IECs). **(D)** The western blot analysis of pSTAT3, STAT3 in Non-SI epithelial cells.

It has been reported that the short-chain fatty acids (SCFAs), including butyrate, can activate downstream JAK/STAT (28), p38 (29), NF-κB (30), PI3K/AKT (31), Wnt/β-catenin (32), and other signaling pathways to promote intestinal mucosa proliferation and repair. *B. fragilis* increased the abundance of the genera of the butyrate-producing *Lachnospiraceae*_NK4A136_group, which are beneficial for intestinal mucosa regeneration. Next, we attempted to explore the underlying mechanism of *B. fragilis* in the intestinal mucosa repair after radiation injury. Thus, we detected some classic proliferation pathways including signal transducer and activator of transcription (STAT3) (33), pmTOR, pNF-κB, and p38 signaling. The western blot results showed that *B. fragilis* activated STAT3 phosphorylation in the intestinal tissue, while *B. fragilis* did not affect the other classic proliferation pathways, such as pmTOR, pNF-κB, and p38 (Figure 5A). Meanwhile, we found that *B. fragilis* increased the expression of the proliferation index PCNA, goblet cell marker MUC2, stem cell indicator Lgr5, and tight junction protein claudin-1 (Figure 5A).

The regulation of STAT3 is complex, as it is involved in signal transduction pathways in numerous cell types under various conditions. Pickert et al. (34) reported that interleukin (IL)-22 motivated STAT3 signaling pathway in IECs to repair mucosal wound in mice with experimental colitis. Notably, STAT3 phosphorylation in immune cells may exert different inflammation effects in colitis (35–37). We attempted to identify the specific layer in the small intestines where STAT3 phosphorylation is triggered after *B. fragilis* administration in radiation-induced intestinal injury. We found that *B. fragilis* mainly promoted STAT3 phosphorylation in the SI IECs but not in the non-epithelial layer (Figures 5B–D), suggesting that *B. fragilis* upregulated the STAT3 signaling pathway in SI IECs. It suggested that *B. fragilis* activated STAT3 phosphorylation with the upregulation with PCNA, MUC2, Lgr5, and claudin-1 in SI IECs.

### *Bacteroides fragilis* promoted slower intestinal intestinal epithelial cell proliferation, stem cell regeneration, goblet cell secretion, and tight junction repair through STAT3 signaling pathway

**FIGURE 6:**
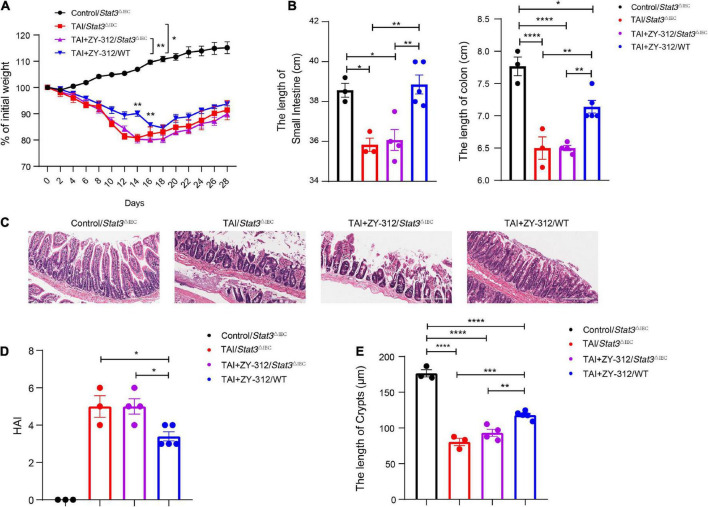
**(A)** The *p*-value between groups are following: percent (%) of weight loss *p*-value (TAI+ZY-312/*Stat3*^△IEC^ vs. TAI+ZY-312 group/WTgroup), Day14 0.0059 and Day16 0.0037. **(B)** The *p*-value between groups are following: *p*-value the small intestine length the colon length, (Control/*Stat3*^△IEC^ group vs. TAI/*Stat3*^△IEC^ group) 0.0171 <0.0001, (Control/*Stat3*^△IEC^ group vs. TAI+ZY-312/*Stat3*^△IEC^ group) 0.02 <0.0001, (TAI/*Stat3*^△IEC^ group vs. TAI+ZY-312/WT group) 0.0041 0.009, and (TAI+ZY-312/*Stat3*^△IEC^ group vs. TAI+ZY-312/WT group) 0.0041 0.005. **(C)** The statistical analysis of colon length. **(D)** The *p*-value between groups are following: *p*-value HAI (TAI/*Stat3*^△IEC^ group vs. TAI+ZY-312/WT group) 0.0372 and (TAI+ZY-312/*Stat3*^△IEC^ group vs. TAI+ZY-312/WT group) 0.0232. **(E)** The *p*-value between groups are following: *p*-value SI Crypts (Control/*Stat3*^△IEC^ group vs. TAI/*Stat3*^△IEC^ group) <0.0001, (Control/*Stat3*^△IEC^ group vs. TAI+ZY-312/*Stat3*^△IEC^ group) <0.0001, (Control/*Stat3*^△IEC^ group vs. TAI+ZY-312/WT group) <0.0001, (TAI/*Stat3*^△IEC^ group vs. TAI+ZY-312/WT group) 0.0003, and (TAI+ZY-312/*Stat3*^△IEC^ group vs. TAI+ZY-312/WT group) 0.0041. The length of SI crypts between groups. Control group/Stat3^ΔIEC^ (*N* = 3), TAI/Stat3^ΔIEC^group (*N* = 3), TAI *+* ZY-312/Stat3^ΔIEC^ group (*N* = 4), TAI *+* ZY-312/WT group (*N* = 5). The data are presented as mean ± SEM, and *p < 0.05, **p < 0.01, **p < 0.001, **p <0.0001.

To verify whether *B. fragilis* promoted SI IECs regeneration through the STAT3 signaling pathway, we constructed a radiation-induced intestinal injury model in *Stat3*^△IEC^ (STAT3 defects in SI epithelium) mice and administered the mice *B. fragilis* orally (Supplementary Figure 1). Compared with the *Stat3*^△IEC^ mice receiving *B. fragilis* (TAI + ZY-312/*Stat3*^△IEC^ group), wild-type mice receiving *B. fragilis* (TAI + ZY-312/WT group) had significantly lower weight loss (Figure 6A), slower intestinal (SI and colon) length shortening (Figure 6B), attenuated SI epithelial cells damage (Figure 6C), reduced overall HAI (Figure 6D), and had longer lengths of SI crypts (Figure 6E). This suggested that *B. fragilis* relieved radiation-induced intestinal injury through the STAT3 signaling pathway in SI IECs.

**FIGURE 7:**
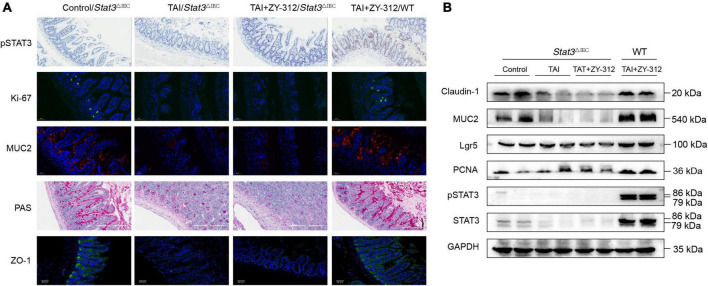
*Bacteroides fragilis* promoted intestinal epithelial cell proliferation, stem cell regeneration, goblet cell secretion, and tight junction repair through the signal transducer and activator of transcription 3 (STAT3) signaling pathway. **(A)** The histological staining of pSTAT3, Ki-67, MUC2, periodic acid-Schiff stain (PAS), and ZO-1 in SI IECs. **(B)** The western blot analysis of pSTAT3, STAT3, MUC2, Claudin-1, Lgr5, and PCNA in intestinal epithelial cell (SI) intestinal epithelial cell (IECs) between groups. GAPDH was regarded as an internal reference.

We found that SI IECs in *Stat3*^△IEC^ mice after abdominal radiation injury had a very low expression of pSTAT3, while the SI IECs from wild-type mice receiving *B. fragilis* after abdominal radiation injury had a higher expression of pSTAT3 (Figure 7A). In this context, we discovered that mice receiving *B. fragilis* in WT mice (TAI + ZY-312/WT group) upregulated the expression of PCNA, MUC2, Lgr5, ZO-1, and claudin-1 (Figures 7A,B), while mice receiving *B. fragilis* in *Stat3*^△IEC^ mice (TAI + ZY-312/*Stat3*^△IEC^ group) showed little expression of the markers above (Figures 7A,B). This suggested that *B. fragilis* requires the STAT3 signaling pathway to promote SI IECs proliferation, stem cell regeneration, mucus secretion, and tight junction proteins expression.

**FIGURE 8:**
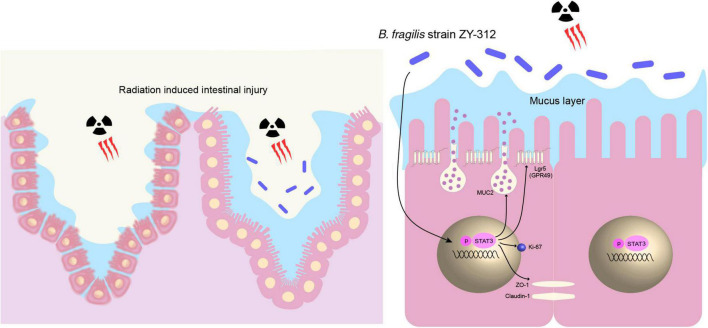
*B. fragilis* promoted intestinal barrier integrity through the STAT3 signaling pathway in a mouse model of radiation-induced intestinal injury. *B. fragilis* promoted SI IEC proliferation, stem cell regeneration, goblet cell secretion, and tight junction repair through the STAT3 signaling pathway hence relieving radiation-induced intestinal injury in mice.

Overall, our results showed that *B. fragilis* promoted intestinal barrier integrity through the STAT3 signaling pathway in a mouse model of radiation-induced intestinal injury (Figure 8).

## Discussion

Gut microbiota dysbiosis plays a crucial role in the pathogenesis of radiation-induced intestinal injury. The abundance of most bacteria belonging to the phyla *Actinobacteria* and *Proteobacteria* has increased, while that of microorganisms from *Firmicutes* and *Bacteroides* has decreased (38, 39). This suggests that the replenishment of *Firmicutes* and *Bacteroides* play protective roles in restoring the gut microbiota structure and relieving radiation-induced intestinal injury. Cui et al. (40) reported that fecal microbiota transplantation could ameliorate radiation-induced toxicity and improve the prognosis of patients after radiotherapy. Furthermore, a randomized, double-blind, controlled trial showed that *Lactobacillus acidophilus* LAC-361 and *Bifidobacterium longum* BB-536 belonging to the phylum *Firmicutes* decreased diarrhea caused by radiation enteritis (41, 42). However, the effect and mechanism of the probiotic *B. fragilis*, belonging to the phylum *Bacteroides*, on radiation-induced intestinal injury remains unclear.

In this study, we found that *B. fragilis* promoted IEC repair to relieve radiation-induced intestinal injury in mice. It was similar to a study that reported that *L. rhamnosus* promoted IEC survival in the crypt region of mesenchymal stem cells and reduced epithelial cell apoptosis in radiation-induced intestinal injury (43). The intestinal barrier is the first defense line in the gastrointestinal tract, which consists of a mucus layer and a subjacent epithelium monolayer with tight junction proteins (44, 45). At the bottom of crypts, intestinal stem cells (ISCs) differentiate into goblet cells and promote epithelial regeneration against radiation-induced intestinal injury (46). It has been reported that the mucus secreted from goblet cells performs an important role in radiation-induced intestinal injury (47). While at the top of crypts, the most important intercellular tight junction is mainly composed of claudin and ZO-1, which exert a vital role in sustaining cell polarity and the intestinal epithelial barrier (48, 49). A clinical study reported that patients receiving radiotherapy had increased intestinal permeability and tight junction disruption (50). Besides, the following radiotherapy patients express limited amounts of tight junction proteins in the intestinal epithelium (51). It suggests that the level of stem cells, goblet cells, and tight junction may be potential markers of the degree of intestinal epithelium repair after abdominal radiation. In our study, we found that *B. fragilis* promoted IEC proliferation, stem cell regeneration, goblet cell secretion, and tight junction repair in radiation-induced intestinal injury. It was reported that a part of the symbiotic microbiota resides in the mucus layer to absorb the nourishment, and mucus has a scouring effect on pathogenic bacteria to relieve colitis (52). Based on the results that *B. fragilis* increased mucus expression in radiation-induced intestinal injury, it suggests that *B. fragilis* may have an influence on intestinal microbiota through mucus secretion. In this study, we analyzed the gut microbiota in mice through 16S rRNA gene sequencing. we found that *B. fragilis* strain ZY-312 did not lead to a significant increase in the abundance of phylum *Firmicutes* as well as the bacterial alpha and beta diversity. However, It is noteworthy that *B. fragilis* increased the general abundance of the *Lachnospiraceae*_NK4A136_group, which are butyrate-producing bacteria that are beneficial for intestinal mucosa protection, immune regulation, and inflammation inhibition (53). In addition, *B. fragilis* increased the abundance of the *Clostridiales*_vadinBB60_group in LEfSe analysis, which was related to the rise of vitamin D in the human body after ultraviolet b (UVB) radiation exposure (54). *B. fragilis* has no significant influence on the intestinal microbiota after radiation-induced intestinal injury in mice, it suggests that *B. fragilis* may promote intestinal epithelium repair through underlying molecular-mediated mechanisms. In the study, we found that *B. fragilis* motivated the STAT3 phosphorylation in SI IECs, it indicated that *B. fragilis* required the participation of the STAT3 signaling pathway to promote intestinal mucosa regeneration.

STAT3 plays an important role in the intracellular signal transduction pathway for cell proliferation and differentiation (55). The phosphorylation of STAT3 will translocate to the nucleus and activates downstream genes involved in proliferation and mucosa defense (56). It has been reported that the motivation of the STAT3 signaling pathway along the crypt-villus axis of the intestinal epithelium is mainly involved in the repair response to damage (57). And STAT3-disrupted intestinal organoids were unable to survive after suffering irradiation (58). It suggests that STAT3 is required for intestinal mucosa regeneration after radiation damage. STAT3 participates in intestinal epithelial cell regeneration including stem cells and goblet cell proliferation, and tight junction repair. STAT3 is an important regulator of intestinal epithelial stem cells (59). And probiotics have the capacity to motivate the STAT3 signaling to promote repair after damage. Yu et al. revealed that the probiotic *Lactobacillus* could activate STAT3 signaling to promote stem cell proliferation in DSS-induced colitis model mice (60). Another study reported that the upregulation of the STAT3 signaling pathway is related to mucus secretion in mice with experimental colitis (61). In addition, the activation of the STAT3 signaling pathway increased the expression of the tight junction protein ZO-1 and occludin (62). In our study, we found that *B. fragilis* promoted intestinal epithelial cell proliferation, stem cell regeneration, goblet cell secretion, and tight junction repair through the STAT3 signaling pathway. It is consistent with those studies that support the feasible relationship between the upstream STAT3 signaling pathway and the downstream activity of IECs including stem cells, goblet cells, and tight junctions.

In conclusion, our results suggested that the probiotic *B. fragilis* promoted SI IEC proliferation, stem cell regeneration, goblet cell secretion, and tight junction repair through the STAT3 signaling pathway in a mouse model of radiation-induced intestinal injury. In our next study, we will further explore the underlying mechanisms through which *B. fragilis* mediates intestinal metabolism and immunity. Our results may provide new insights for its application as a functional and clinical drug for radiation-induced intestinal injury after radiotherapy.

## Conclusion

*Bacteroides fragilis* strain ZY-312 promoted small intestinal epithelial cell proliferation, stem cell regeneration, goblet cells secretion, and tight junction repair through the STAT3 signaling pathway in a mouse model of radiation-induced intestinal injury.

## Data availability statement

The original contributions presented in this study are publicly available. The data presented in the study are deposited in the Figshare repository, accession number: doi: 10.6084/m9.figshare.21310536.

## Ethics statement

This animal study was reviewed and approved by L2018053.

## Author contributions

QZ was responsible for bacteria strain culture, animal experiment, data analysis, and manuscript writing. BS, RH, HL, WZ, MS, KL, XL, and SC were in charge of molecular detection. YL and YW provided the *B. fragilis* strain ZY-312, revised the manuscripts, and offered financial assistance. FZ was responsible for the project design and acquired funding. All authors contributed to the article and approved the submitted version.
