# Supplementary material for: Bacteroides fragilis strain ZY-312 promotes intestinal barrier integrity via upregulating the STAT3 pathway in a radiation-induced intestinal injury mouse model
Source: Front Nutr. 2022 Dec 15;9:1063699. doi: 10.3389/fnut.2022.1063699 (PMC9798896; doi:10.3389/fnut.2022.1063699)
Supplement: Supplementary file 1 [file Data_Sheet_1.docx]

*Bacteroides fragilis* strain ZY-312 promotes intestinal barrier integrity via upregulating the STAT3 signaling pathway in a radiation-induced intestinal injury mouse model

Qian Zhou^1^, Binhai Shen^1^, Ruo Huang^1^, Hongbin Liu^1^, Wendi Zhang^1^, Mengyao Song^1^, Ke Liu^1^, Xinlong Lin^1^, Shuze Chen^1^, Yangyang Liu^2^, Ye Wang^2^, Fachao Zhi^1*^.

^1^Guangdong Provincial Key Laboratory of Gastroenterology, Institute of Gastroenterology of Guangdong Province, Department of Gastroenterology, Nanfang Hospital, Southern Medical University, Guangzhou 510515, China

^2^Guangzhou ZhiYi Biotechnology Co., Ltd., Guangzhou 510535, China

*** Correspondence: Fachao Zhi, zhifc41532@163.com.**

**ORCID:https://orcid.org/0000-0001-8674-4737.**

**DOI:** [**https://figshare.com/s/ca923b085485ef71a75f**](https://figshare.com/s/ca923b085485ef71a75f)

# Supplementary Material

## FIGURE S1 | WT mice received relieved radiation-induced intestinal injury. (A)The weight loss. (B)The statistical analysis of SI and colon length. (C) The statistical analysis of HAI. (D) The PAS staining of SI. Control group/WT (N=3), TAI/WT group (N=5). The data are presented as mean ± SEM, and *p < 0.05, **p < 0.01, **p < 0.001, **p < 0.0001.

## Table S1. Detailed scoring method of histopathology associated index.

| Scores | Colonic epithelial damage | Inflammatory cell infiltration | | |
| --- | --- | --- | --- | --- |
|  |  | mucous layer | submucous layer | seromuscular layer |
| 0 | Normal | Normal | Normal | Normal |
| 1 | Hyperplasia, irregular crypts, and goblet cells disappeared | mild | mild to moderate | moderate to severe |
| 2 | Medium and mild crypt disappeared(10-50%) | moderate | severe | / |
| 3 | Severe crypts disappear(50-90%) | severe | / | / |
| 4 | The crypt disappeared completely without ulcer formation | / | / | / |
| 5 | Small to medium ulceration (ulcer width< 10 crypt width) | / | / | / |
| 6 | Large ulcer (ulcer width ≥10 crypt width) | / | / | / |

## Table S2.Primers of Gene knockout mouse model were used in reverse transcriptase polymerase chain reaction.

| Primer Name | Sequence（5’-3’） |
| --- | --- |
| Vil1-ProF1 | GTGTTTGGTTTGGTTTCCTCTGCATAAGA |
| Cre5R1 | GCAGGCAAATTTTGGTGTACGGTCA |
| 19436 | TTGACCTGTGCTCCTACAAAAA |
| 19437 | CCCTAGATTAGGCCAGCACA |

# Supplementary methods

## Preparation of Bacteroides fragilis ZY-312-derived capsular polysaccharide A

(1) Take 50g of bacteria mud, add 300g purified water to make bacteria weight suspension, adjust its pH to 3.5 with 1mol/L hydrochloric acid solution, extract 1.5h at 100℃, cool to room temperature, centrifuge 12000g at room temperature for 10min, take supernatant, get crude sugar solution;

(2) The crude sugar solution was concentrated by ultrafiltration with a 10KD ultrafiltration membrane, and small molecular impurities were removed until the conductivity was stable, and the reflux solution was collected;

(3) An equal volume of 40mmol/L Tris-HCl (pH8.5) was added to the reflux solution. DEAE Sepharose Fast Flow ion exchange column chromatography (16mm×200mm), the flow rate was 20mL/min, 20mmol/L Tris-HCl (pH8.5, containing 0.2mol/L NaCl) gradient elution was performed on 25 column volumes and collected in sections. 100mL/ bottle (component), SEC-HPLC tracking monitoring, combined with 206nm of the component with a single peak and symmetrical peak, 10KD ultrafiltration membrane ultrafiltration, purified water was added repeatedly ultrafiltration until the conductivity was stable, the reflux solution was collected, lyophilized, *Bacteroides fragilis* extract was obtained.

(4) Weigh 30mg of *Bacteroides fragilis* extract described in step (3), dissolve in 0.5mL D2O, and add 1μl acetone (1H, 2.22; 13C, 30.89) calibration. 1H, 13C, COSY, HSQC, and HMBC spectra were analyzed by a 500 MHz Bruker NMR spectrometer (see Figure 3A-E). The extracts collected in step (3) were capsular polysaccharide A, with binding lipid content less than 0.02%, protein residue less than 1%, and nucleic acid residue less than 0.05%. By GPC (gel permeation chromatography) analysis, the average weight of polysaccharide A was 80-90kDa, Mw/Mn was 1.0-1.3, and the proportion of Mw distributed in 70KD-100KD accounted for 70-80% of the total.
