# Supplementary figures and images for: Bacteroides fragilis strain ZY-312 promotes intestinal barrier integrity via upregulating the STAT3 pathway in a radiation-induced intestinal injury mouse model
Source: Front Nutr. 2022 Dec 15;9:1063699. doi: 10.3389/fnut.2022.1063699 (PMC9798896; doi:10.3389/fnut.2022.1063699)

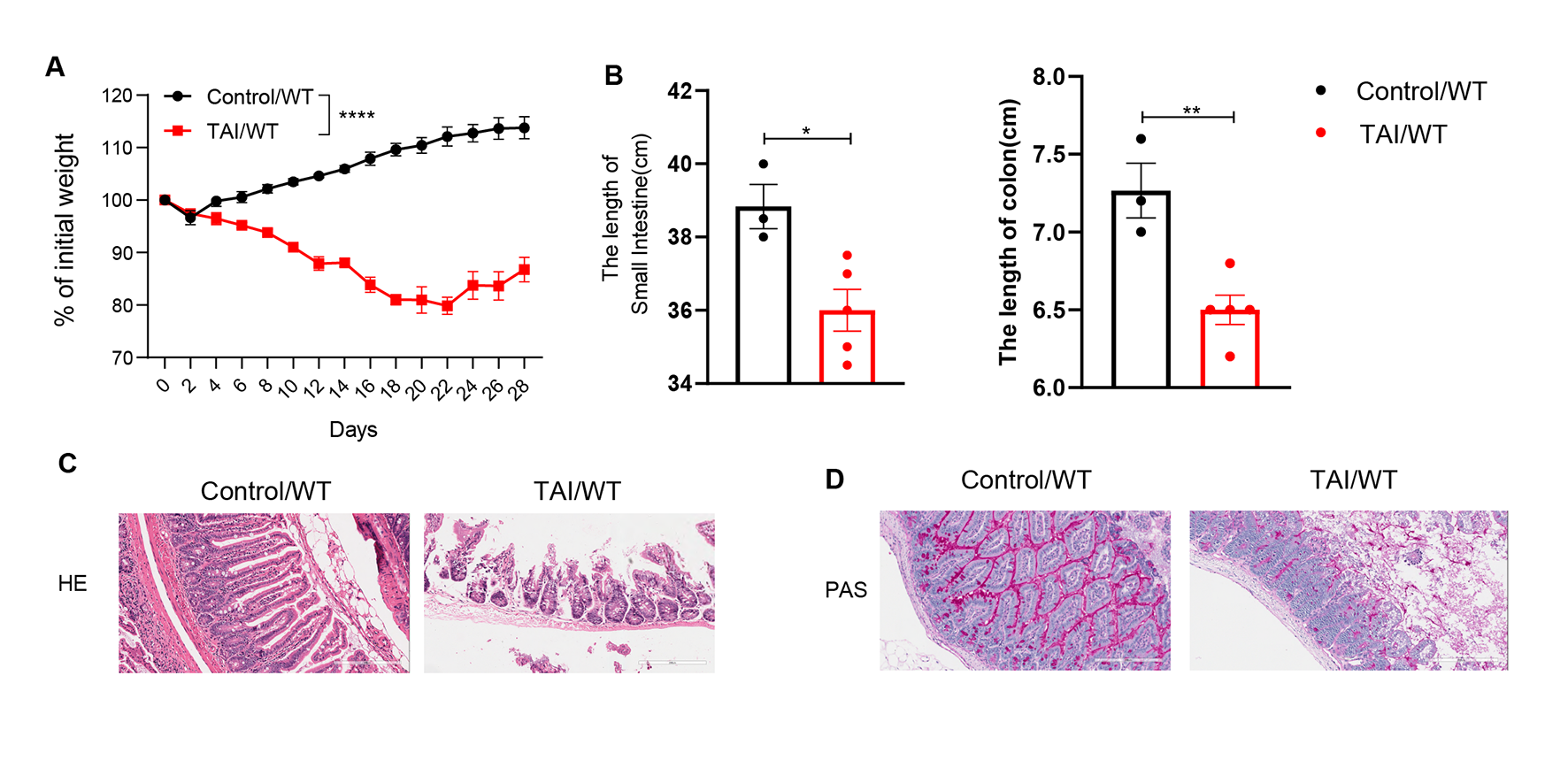

Supplement: Supplementary file 2 [file Image_1.TIF]
